# Characterizing a sexual health and HIV risk stratification scale for sexually active adolescent girls and young women (AGYW) in Tanzania

**DOI:** 10.1371/journal.pone.0248153

**Published:** 2021-03-18

**Authors:** Hannah Han, Fan Yang, Sarah Murray, Gaspar Mbita, Maggie Bangser, Katherine Rucinski, Albert Komba, Caterina Casalini, Mary Drake, Esther Majani, Kelly Curran, Yeronimo Mlawa, Agnes Junga, Jeremie Zoungrana, Upendo Kategile, Angela Ramadhani, Qian-Li Xue, Stefan Baral

**Affiliations:** 1 Department of Epidemiology, Johns Hopkins Bloomberg School of Public Health, Baltimore, United States of America; 2 Department of International Health, Johns Hopkins Bloomberg School of Public Health, Baltimore, United States of America; 3 Department of Mental Health, Johns Hopkins Bloomberg School of Public Health, Baltimore, United States of America; 4 Jhpiego, Dar es Salaam, Tanzania; 5 Independent Consultant, New York, United States of America; 6 Jhpiego, Baltimore, United States of America; 7 Engender Health, Dar es Salaam, Tanzania; 8 United States Agency for International Development, Dar es Salaam, Tanzania; 9 Ministry of Health, Community Development, Gender, the Elderly and Children, Tanzania; 10 Department of Medicine, Johns Hopkins Bloomberg School of Public Health, Baltimore, United States of America; 11 Department of Biostatistics, Johns Hopkins Bloomberg School of Public Health, Baltimore, United States of America; 12 Department of Health Policy and Management, Johns Hopkins Bloomberg School of Public Health, Baltimore, United States of America; University of the Witwatersrand, SOUTH AFRICA

## Abstract

Adolescent girls and young women (AGYW) aged 15 to 24 years face disproportionately high risks of acquiring HIV and other sexually transmitted infections (STIs). A sexual health risk stratification tool can support the development and implementation of tailored HIV and STI prevention services for sub-groups of at-risk AGYW. Data were collected among sexually active AGYW aged 15 to 24 years in Tanzania between April 2015 and March 2017. Exploratory and confirmatory factor analyses were conducted to construct and assess the latent structure of a ten-item scale for rapid assessment of sexual health risks. Items with high factor loadings and minimal cross loadings were retained in the final scale. Scale performance was appraised against condomless sex (defined as unprotected vaginal or anal intercourse) reported by AGYW for construct validity. A three-factor structure of vulnerability to HIV among AGYW was supported with subscales for socioeconomic vulnerability; lack of adult support; and sexual behavioral risks. The chi-square goodness-of-fit test, root mean square error of approximation, comparative fit index, and Tucker-Lewis index indicated a strong goodness-of-fit of the three-factor scale. Cronbach alphas (0.55 for socioeconomic vulnerability, 0.55 for lack of support, and 0.48 for sexual risk) indicated sub-optimal internal consistency for all sub-scales. The factor-item and factor-factor correlations identified in these analyses were consistent with the conceptual framework of vulnerability of HIV infection in AGYW, suggesting good construct validity. The scale also demonstrated a statistically significant association with condomless sex and could be potentially used for sexual health risk stratification (OR = 1.17, 95% CI: 1.12, 1.23). The sexual health and HIV risk stratification scale demonstrated potential in identifying sexually active AGYW at high risk for HIV and other STIs. Ultimately, all AGYW in Tanzania are not at equal risk for HIV and this scale may support directing resources towards those at highest risk of HIV.

## Introduction

Despite the ongoing effort to reduce the global burden of human immunodeficiency virus (HIV) and other sexually transmitted infections (STIs), adolescent girls and young women (AGYW) continue to face a disproportionately high incidence and burden of HIV and other STIs [1,2]. Discrepancies in HIV risk by sex are particularly prominent in sub-Saharan Africa, where 79% of new infections among 10–19 year-olds are in girls [3]. Chlamydia, gonorrhea, and syphilis are also common among AGYW in this region, which exacerbate their risk of HIV infection [4,5].

Beyond biological factors, gender differences in the burden of HIV and other STIs among AGYW are driven by behavioral, social, and structural factors that operate together to shape individual vulnerability [6,7]. Structural drivers of engaging in unsafe sexual behaviors among AGYW include poverty, food insecurity, living in informal settlements, and exposure to community violence, including intimate partner violence [7,8]. The relationship between the risk of HIV acquisition and these structural factors could be mediated by individual-level psychosocial factors, such as child abuse and substance abuse [7]. In these contexts, many young women enter transactional and often inter-generational sexual relationships with partners who can provide them financial support and security, and/or increased social status [9]. Such financial dependence and gender norms in age-disparate relationships not only compromise condom negotiation, but also increase risks for forced or coercive sex and unintended adolescent pregnancy [9].

Characterizing the heterogeneity of sexual and reproductive risk across behavioral, social, and structural levels is critical in delivering tailored services to mitigate AGYW’s HIV and STI vulnerability. Particularly in situations of significant resource constraints, the differentiation can help focus sexual health services on those in greatest need [10]. Thus far, a number of instruments have been developed to assess HIV risk levels in different populations. However, very few target AGYW aged 15–24 years, and none of them are designed specifically for out-of-school girls. Out-of-school girls are considered the most vulnerable group among AGYW because they are hard to reach by school-based HIV and sexual and reproductive (SRH) programs [11]. Consequently, there remains an urgent need to develop a brief tool to identify those who are most in need of HIV services.

Tanzania is one of the United States President’s Emergency Plan for AIDS Relief (PEPFAR) priority countries, where the HIV prevalence among AGYW exceeds 2% [12]. Previous studies reported that AGYW in Tanzania were at heightened risk of sexual exploitation, STIs, and unintended pregnancies [9,13]. A study conducted among pregnant adolescents in Tanzania showed an alarming STI prevalence of approximately 50% [14]. Herpes Simplex Virus Type 2 was the leading contributor of the STI prevalence in this population, which was followed by trichomoniasis and chlamydia. The study also found that being in an age-disparate relationship with older men and prior pregnancy history were strongly correlated with testing positive for one or more STIs [14]. There has been little study of sexual risks among AGYW in Tanzania, and SRH education and services have limited coverage across the country [9,13,15].

The Sauti Project is a five-year PEPFAR/USAID-funded project implemented in partnership with the Ministry of Health, Community Development, Gender, Elderly and Children (MoHCDGEC) of Tanzania and the Tanzania Commission for AIDS. Launched in 2014, Sauti offers combination prevention and HIV testing services to individuals at high-risk in selected regions of Tanzania. The Sauti Project is also an implementing partner of PEPFAR’s DREAMS Initiative (Determined, Resilient, Empowered, AIDS-free, Mentored, and Safe) which was designed to improve the overall well-being of AGYW by reducing HIV transmission through provision of comprehensive, evidence-based HIV prevention and treatment packages [16,17]. The DREAMS Initiative has been implemented in nine additional countries in sub-Saharan Africa [16]. To maximize the programmatic impact of Sauti and DREAMS, a new sexual health and HIV risk assessment scale was developed to characterize risk-appropriate services specifically for AGYW aged 15–24 years. The tool was adapted from existing vulnerability indices used in Tanzania and other countries in eastern or southern Africa, such as Go Girls! (GGI) Initiative Vulnerable Girls Indices (Botswana, Malawi, Mozambique) [18,19] and the Adolescent Girls Vulnerability Index (Uganda) [20]. Through the use of exploratory and confirmatory factor analysis (EFA and CFA), this study seeks to refine and evaluate the factor structure of this Sauti tool for measuring AGYW’s vulnerability to HIV and assess the validity of the resulting sexual health and HIV risk stratification scales.

## Materials and methods

### Ethical statement

The National Institute for Medical Research (NMRI) and the Ministry of Health Community Development, Gender, Elderly and Children of the United Republic of Tanzania provided the ethical clearance for the primary data collection of both primary and health program screening data. The sexual health and HIV risk stratification scale was developed as a programmatic tool, which was administered in the context of routine service delivery within the Sauti project. Because data collected were used to guide this routine service delivery, parental consent was only sought for HIV testing if the participant was under the age of 18 in accordance with the law in Tanzania. Minors under the age of 18 who were already parents were considered emancipated, and thus they were able to consent for themselves.

All participants provided verbal informed consent because of extremely low literacy rates among disenfranchised AGYW and also because data were collected in the context of a program where answers to the risk index affected the programs to which people had access as part of a differentiated care model. This was approved by the aforementioned institutional review boards.

The secondary data analysis for this study was approved by the institutional review boards of Johns Hopkins Bloomberg School of Public Health (IRB No 00006673) and the National Institute of Medical Research of Tanzania (Extension number NIMR/HQ/R.8c/Vol. 1/ 678, dated 25th April 2019) as a non-human subject research. This is because the data used for analyses did not include any identifiers and focused on assessing the quality of the scale in terms of its psychometric properties. Primarily, this vulnerability scale was implemented to support quality improvement in the delivery of differentiated HIV prevention services for AGYW in Tanzania.

### Study instrument

For the purpose of this study, *risk* is operationally defined to include a range of individual, interpersonal, and structural factors that influence susceptibility to HIV and other STIs. A multi-staged process was used to develop items that assess sexual health risks faced by sexually-active AGYW aged 15–24. First, a literature search was conducted to extract factors that have been demonstrated to predict teen pregnancy and incident HIV and STIs among AGYW aged 15–24 years. The Sauti Project team then convened a consultation with partners and stakeholders—representing the Tanzania Commission for AIDS (TACAIDS), Prime Minister’s Office—Regional Administration and Local Government (PMO RALG), Ministries of Health and Social Welfare, Home Affairs, and Youth Development in Tanzania, the United States Agency for International Development (USAID), the Centers for Disease Control and Prevention (CDC), the Joint United Nations Programme on HIV/AIDS (UNAIDS), the United Nations International Children’s Emergency Fund (UNICEF), Sauti-project’s team, and both local and international non-profit organizations working with AGYW—to review tools from other countries used to assess AGYW’s HIV risk. The tools included the Go Girls! (GGI) Initiative Vulnerable Girls Indices (Botswana, Malawi, Mozambique) [18,19] and the Adolescent Girls Vulnerability Index (Uganda) [20]. Partners and stakeholders also reviewed several Tanzanian sources including the national OVC (Orphan and Vulnerable Children) Indicators, and other adolescent-related indicators developed by the Tanzania Ministry of Health, UNICEF and the Population Council. Based on these sources, participants at the consultation developed candidate items for the scale. Several iterations provided an opportunity to assemble, review, and revise scale items. Each item was assigned an ascending risk score ranging from 0 to 3, with 0 being no risk and 3 being the highest risk. The scale was then translated into Kiswahili and pilot-tested with 600 AGYW in clinics in Dar es Salaam.

### Study sample

Between April 2015 and March 2017, a total of 10,055 AGYW aged 15–24 years were recruited across mainland Tanzania. Specifically, they were recruited from “hotspots” (i.e., bars, guest houses, salons, market places), service delivery points (i.e., mobile community-based HIV testing and counseling (HTC) centers), social and behavior change communication (SBCC) platforms, and other Sauti Project services. All study participants below age 18 were considered “out-of-school”, which was defined in accordance with the Tanzanian Demographic Health Survey as attending less than ten days of school in the past three months. Trained local interviewers administered the scale in-person in Kiswahili to all participants who consented to participate in the study. AGYW who reported never having had vaginal or anal sex were excluded from the analyses. To accommodate the study aims and design, the study sample was evenly split into derivation (N = 5,027) and validation (N = 5,028) groups using computer-generated randomization. The derivation group was used to develop the risk scale using exploratory factor analysis, and the validation group was used to test the factor structure developed in the derivation group using confirmatory factor analysis.

### Statistical analyses

Socio-demographic and sexual behavioral characteristics of AGYW in both derivation and validation groups were summarized using descriptive statistics, and Wilcoxon rank sum tests or Pearson’s chi-square tests were used to assess for statistically significant differences by group. To explore the underlying factor structure and examine construct validity, EFA was conducted. First, all 20 items in the questionnaire were recoded as binary, with a risk score of 1–3 being categorized as “high risk” and a risk score of 0 being categorized as “low risk.” Items with greater than 5% missingness or poor face validity were dropped. Items related to condom use were not included in the analysis of the scale as we considered those a proxy for HIV infection; condomless sex, whether it is vaginal or anal intercourse, is the most common mode of HIV transmission among adults. The scale score was compared to the responses on these items to assess external construct validity. A total of 15 items were included in EFA and CFA (Table 1).

**Table 1:** Items in the sexual health and HIV risk stratification scale.

Variable	Variable	Question
Item 1*	Anal sex history	Have ever had anal sex
Item 2*	Sexual violence	Have ever experienced sexual violence
Item 3*	Physical violence	Have ever experienced physical violence
Item 4*	Food security	In the past one month, have gone to sleep at night hungry because there was no food
Item 5*	Financial support	Do not have an adult whom I can go for financial support
Item 6*	Emotional support	Do not have an adult whom I can go for emotional support
Item 7*	Age at coital debut	Had first sex at 15 years of age or below
Item 8*	Concurrent sexual partners	Have had concurrent sexual partners in the past 12 months
Item 9*	Transactional sex	Have had sex with anyone because he provided gifts or helped pay for things
Item 10*	Partner’s HIV status	My current partner(s) is HIV-positive or have unknown HIV status
Item 11	Cohabitation	Currently married or living with a man as if married
Item 12	Social isolation	Currently a member of a social group
Item 13	Age-disparate relationships	Have had a sexual partner who is 5+ years older
Item 14	Teen pregnancy history	Had first pregnancy at 15 years of age or below
Item 15	Education	Highest education completed is less than primary school

* Items 1–10 were retained in the final sexual health and HIV risk stratification scale

The Kaiser-Meyer-Olkin (KMO) (>0.7) measure of sampling adequacy was calculated and Bartlett’s Test of Sphericity (p<0.05) was performed on the derivation group to assess if the data were suitable for factor analysis [21]. As items were binary, polychoric correlation matrices were used to estimate the degree of associations between observed variables [22]. Results of a parallel analysis, Kaiser’s criterion [21], eigenvalues, scree plots, Horn’s test, and interpretability of factors were considered in determining the number of factors to retain [23,24]. EFA was conducted using promax oblique rotation [25]. After rotation, factor loadings of all items were examined. Items with a loading within the range of 0.3 and 0.9 on a factor [24] with no or few cross loadings were retained. Internal consistency of each resulting sub-scale was examined using Cronbach’s alpha [26]. Total scale and subscale scores for each participant were then calculated by summing responses across items.

After identifying the internal structure of the scale, CFA was conducted using data from the validation group. A polychoric correlation structure and weighted least squares estimator was used. Goodness-of-fit was examined using the chi-squared (χ^2^) goodness-of-fit test, the general model significance (p), the root mean square error of approximation (RMSEA), the comparative fit index (CFI), and the Tucker-Lewis index (TFI). Model fit was considered acceptable if χ^2^ and p were non-significant (p>0.05), RMSEA was 0.08 or lower, and CTI and TFL were greater than 0.9 [27]. External construct validity was assessed by looking at the association between the scale scores and a behavioral proxy for HIV acquisition, namely condomless sex, using logistic regression. EFA was conducted using Stata 15 [28] and CFA was conducted using Mplus [29,30].

## Results

### Participants’ sociodemographic characteristics

A total of 3,277 and 3,280 participants met the eligibility criteria and were included in derivation and validation groups, respectively. Participants’ socio-demographic and sexual behavior characteristics are presented in Table 2. The median age was 21 years (IQR: 19–23) in both groups, and most of the participants had completed some primary school (54% and 55% in the derivation and validation groups, respectively). One-quarter of the study participants were married at the time of recruitment and about 80% reported sexual debut under the age of 16. More than half of participants in both derivation and validation groups reported not having used condoms during their last three vaginal and/or anal sex. Sociodemographic characteristics were comparable in both groups, suggesting randomization was successful in achieving balanced groups.

**Table 2:** Baseline socio-demographic and behavioral characteristics of adolescent girls and young women aged 15–24 years.

Variables	Number of participants (%)	
	Derivation group (N = 3,277)	Validation group (N = 3,280)
Median age (IQR)	21 (19, 23)	21 (19, 23)
Education		
→Never attended school	166 (5%)	189 (6%)
→Incomplete primary school	409 (12%)	390 (12%)
→Completed primary school	1,391 (42%)	1,399 (43%)
→Incomplete secondary school	589 (18%)	588 (18%)
→Completed secondary school	722 (22%)	714 (22%)
Marital status		
→Not married	2,410 (74%)	2,458 (75%)
→Married	867 (26%)	822 (25%)
Have gone to bed hungry because no food		
→None	2,237 (68%)	2,201 (67%)
→Rarely	352 (11%)	385 (12%)
→Sometimes	192 (6%)	232 (7%)
→Often	222 (7%)	207 (6%)
→Do not know how often	274 (8%)	255 (8%)
Sexual history		
→Only had vaginal sex	3,125 (95%)	3,127 (95%)
→Had both vaginal and/or anal sex	152 (5%)	153 (5%)
Age at sexual debut		
→≤15 years old	2,610 (80%)	2,577 (79%)
→16–18 years old	647 (20%)	574 (21%)
→> 18 years old	20 (1%)	28 (1%)
Pregnancy history		
→Never been pregnant	1,571 (48%)	1,578 (48%)
→Have been pregnant	1.706 (52%)	1,702 (52%)
Age at pregnancy		
→<15 years old	87 (5%)	93 (5%)
→15–18 years old	473 (28%)	505 (30%)
→> 18 years old	1,065 (62%)	1,035 (63%)
→Don’t know when	82 (5%)	69 (2%)
Condom use in last 3 vaginal sex		
→None	1,686 (51%)	1,710 (52%)
→Once	471 (14%)	450 (14%)
→Twice	390 (12%)	389 (12%)
→Three times	510 (16%)	513 (16%)
→Don’t know	212 (6%)	206 (6%)
Maximum number of concurrent sexual partners in the last 12 months		
→None	2,104 (64%)	2,052 (63%)
→≥3 sexual partners	391 (12%)	408 (12%)
→two sexual partners	667 (20%)	706 (22%)
→Don’t remember number	115 (4%)	114 (3%)

### Exploratory factor analysis

The Kaiser-Meyer-Olkin statistic (KMO = 0.772) and Bartlett’s test (χ^2^ = 4270.5, p<0.001) indicated that data were well-suited for factor analysis. Iterated principal factor with promax oblique rotation identified a three-factor solution that explained 62% of total variance. Five items–cohabitation, social isolation, age-disparate relationships, teen pregnancy history, and education level–were excluded from the final scale due to either too high (>0.9) or too low factor loadings (<0.3), cross-loading on more than one factor with the similar strength, or low item-test correlations.

Exploratory factor loadings for items in the final scale are presented in Table 3. Factor loadings of the ten items ranged from 0.36 to 0.82. The first factor, called socioeconomic vulnerability, included four items. Cronbach’s alpha for the socioeconomic vulnerability sub-scale was 0.55. The lack of adult support and sexual behavioral factors included two and four items with Cronbach’s alphas of 0.55 and 0.48, respectively. The item-test correlations ranged from 0.29–0.63. The item-rest correlations ranged from 0.14 to 0.44, with the lowest in item 7. All three factors showed optimal correlations (Table 4).

**Table 3:** Exploratory factor analysis loadings of the ten items retained in the final scale using the derivation group.

		Factor loadings		
		Factor 1: Socio-economic vulnerability	Factor 2: Lack of adult support	Factor 3: Sexual behaviors
Item 1	Anal sex history	**0.450**	0.089	0.0650
Item 2	Sexual violence	**0.686**	0.036	0.063
Item 3	Physical violence	**0.825**	-0.133	0.072
Item 4	Food security	**0.503**	0.264	0.070
Item 5	Financial support	-0.059	**0.747**	0.114
Item 6	Emotional support	-0.005	**0.820**	-0.134
Item 7	Age at coital debut	-0.081	-0.035	**0.382**
Item 8	Concurrent sexual partners	0.098	-0.063	**0.806**
Item 9	Transactional sex	0.163	0.038	**0.645**
Item 10	Partner’s HIV status	-0.147	0.183	**0.364**

**Table 4:** Correlation matrix between 3 factors retained in the final scale in derivation group.

	Factor 1	Factor 2	Factor 3
Factor 1	1		
Factor 2	0.444	1	
Factor 3	0.515	0.519	1

### Confirmatory factor analysis

**Fig 1:**
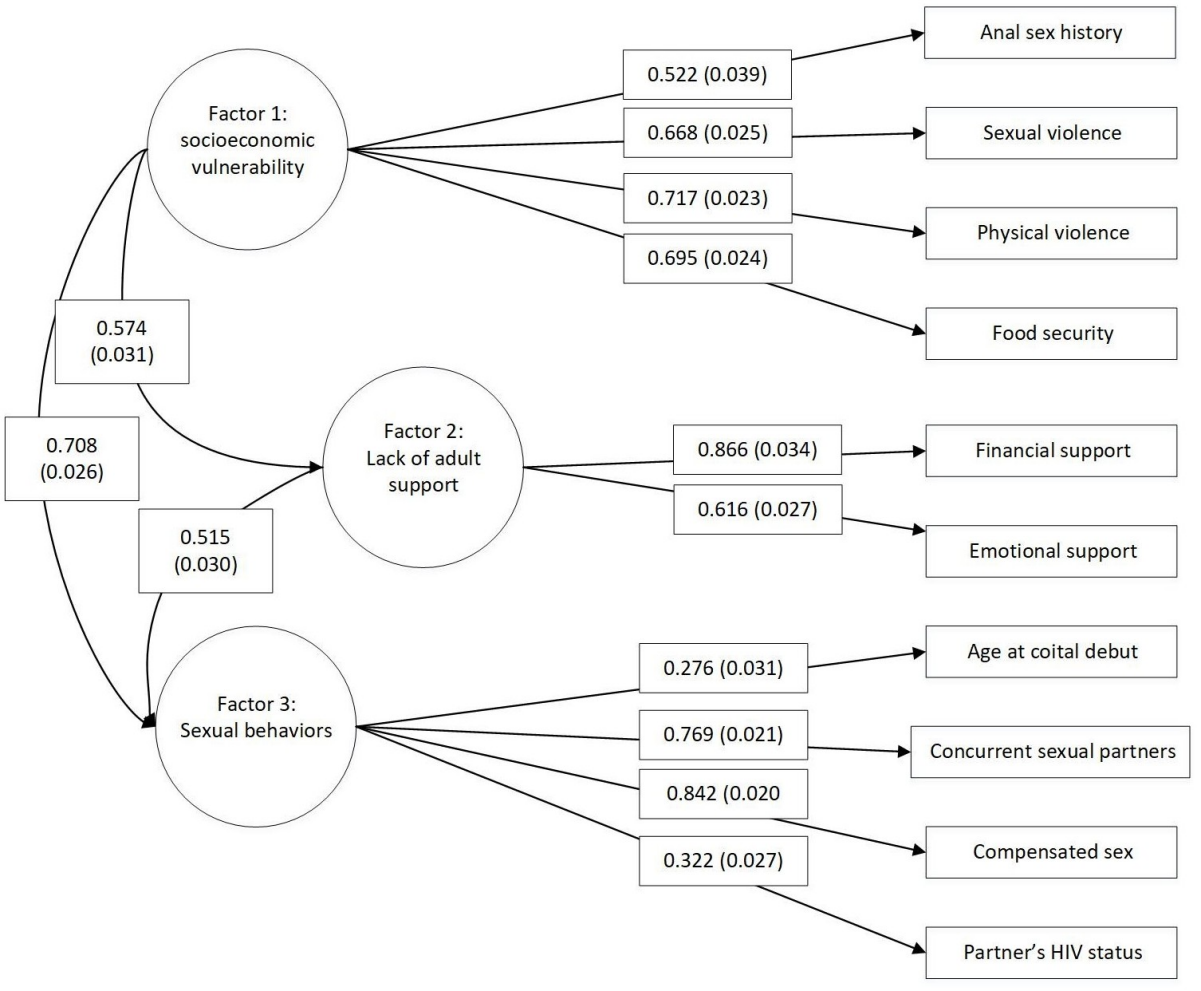
A confirmatory factor analysis of the sexual health and HIV risk stratification scale.

The CFA model fit indices of the three-factor model identified in EFA are shown in Table 5. RMSEA was 0.038, suggesting satisfactory goodness of fit. CFI and TLI were 0.971 and 0.959, respectively, both greater than 0.9 and falling into the acceptable category. In the final three-factor model, item loadings ranged from 0.276 to 0.866 as shown in Fig 1. All item loadings were significant with p-values less than 0.001. The three factors showed statistically significant correlations among each other (F1& F2: 0.574, F1&F3: 0.708, F2&F3: 0.515).

**Table 5:** Goodness of fit indices for confirmatory factor analysis on derivation and validation sets.

	Validation group
χ^2^	180.351
df	23
P	<0.001
RMSEA	0.038
CFI	0.971
TFI	0.959

χ^2^ chi-square goodness-of-fit test, *df* degrees of freedom, *p* general model significance, *RMSEA* root mean square error of approximation, *CFI* comparative fit index, *TFI* Tucker-Lewis index

In validating the scale, final scores were summed up by factors and in total. The scores were appraised against condomless sex. Logistic regression results were presented in Table 6. A one-unit increase in socioeconomic vulnerability (F1) scores was associated with a 12% increase in odds of condomless sex (OR: 1.12, 95% CI: 1.01, 1.24). A one-unit increase in lack of adult support (F2) factor scores was associated with 42% increase in odds of condomless sex (OR: 1.42, 95% CI: 1.24, 1.51). One-unit increase in sexual behavioral risk (F3) factor scores was associated with 35% increase in odds of condomless sex (OR: 1.35, 95%CI: 1.24, 1.47). Each unit increase in the total score was associated with a 17% increase in odds of condomless sex (OR: 1.17, 95% CI: 1.12, 1.23). All regression results were statistically significant with p values <0.05.

**Table 6:** Associations between risk scores and condomless sex among AGYW.

	Odds Ratio	p	95% CI
Total scale	1.17	<0.001	(1.12, 1.23)
Factor 1: Socio-econ vulnerability	1.12	<0.050	(1.01, 1.24)
Factor 2: Lack of adult support	1.42	<0.001	(1.24, 1.61)
Factor 3: Sexual behavioral risk	1.35	<0.001	(1.24, 1.47)

## Discussion

We examined the psychometric properties of a sexual health and HIV risk stratification scale. EFA and CFA results in derivation and validation samples suggested three underlying constructs of HIV risk among AGYW: socioeconomic vulnerability, lack of adult support, and sexual behaviors. This finding highlights the multidimensional nature and heterogeneity of sexual and reproductive health vulnerabilities experienced by sexually active AGYW living in Tanzania. Internal consistency of the sub-scales for each of these constructs was suboptimal in part due to the low number of included items on each scale. The overall scale demonstrated adequate internal consistency and all three sub-scales were significantly associated with higher likelihood of engagement in condomless sex, supporting their external construct validity.

The first factor, socioeconomic vulnerability, explored AGYW’s underlying vulnerability to HIV pertaining to poverty and gender-based power imbalances in sexual relationships [2,31]. Gender disparities observed in many communities across Tanzania have resulted in girls and women, married or not, depending heavily on men for financial and material support [31]. This unequal economic power between genders often perpetuates internalization of oppressive gender norms in a sexual relationship, exposing girls and young women to higher risk of sexual—vaginal and/or anal—and physical violence [32–37]. Extreme poverty could further exacerbate power inequality in sexual relationships and AGYW’s ability to seek appropriate care potentiating the risks for HIV and STIs [35,38–40]. The four items that loaded onto this factor—food insecurity, sexual violence, physical violence, and anal sex practices—collectively demonstrated this broad contextual fabric that make up structural HIV vulnerability of AGYW in Tanzania.

Limited adult support represented the second dimension of Tanzanian AGYW’s vulnerability to HIV. Notably, items related to emotional and financial support from adults loaded onto a distinct factor separate from the socioeconomic vulnerability factor (Factor 1). This suggests a unique HIV vulnerability pathway related to insufficient support at the household- or community-level possibility due to orphanhood or limited support from caregivers. Several studies have shown parenting and family structure could have a significant impact on young people’s self-confidence and inter-relational skills, which in turn affects their sexual and reproductive health [41,42,43,44]. These findings together supported the second dimension of vulnerability emerging from analyses in this study. It highlights the need for differentiated social and psychological care packages for orphaned or neglected AGYW to reduce their advance in the risk pathway and ultimately preventing HIV acquisition. These data also suggest the potential impact of interventions earlier in the risk pathway including through caregiver-oriented interventions to mitigate this dimension of AGYW’s vulnerability to HIV.

The last factor, sexual behaviors, captured more proximal vulnerability to HIV from an epidemiological perspective, which is manifested through items including early sex debut (coital debut below age 15), having HIV seropositive partners, sexual concurrency and transactional sex. The associations between early sex debut, multiple sexual partners, transactional sex and increased risk of HIV and other STIs are well-documented in the literature [45,46,47]. These risk factors pertaining to individuals’ sexual network and behaviors are particularly threatening to out-of-school girls as they typically lack access to resources and interventions from school-based health programs [48,49]. It highlights the importance of targeted, community-based SRH education and services to mitigate their risks early on. This factor is also distinct from the other two factors because it deems to capture the role of female agency in engaging in these sexual behaviors among AGYW. Transactional sex is common among AGYW in Tanzania [50,51]. The motivation for its practice is nuanced and complex, and likely driven by a number of factors, such as survival and social mobility [52,53]. In a relationship, women exercise agency in spite of constraints of patriarchy, poverty, or cultural norms of gender roles [54]. While there is a close link between deprivation and agency, studies have shown that women may be able to exhibit power even in the rural context to select sexual partners and initiate sexual negotiations, including condom negotiation and timing of first sex [47,55,56]. The decision to engage in safe sex, however, varies based on women’s position, expectations and respectability within a given relationship. According to a study conducted in Swaziland, women’s decision in applying risk reduction strategies was often influenced by the potential socioeconomic benefits provided by the relationship even when they were aware of the importance of monogamy and consistent condom use in minimizing HIV risk [57].

Findings from this study should be considered in the context of several limitations. First, this study was conducted cross-sectionally using condomless sex as a proxy measure for HIV acquisition to assess construct validity. While these results provide some support for construct validity, longitudinal analysis using HIV sero-conversation as an outcome would provide stronger evidence for the scales’ validity. We also observed sub-optimal internal consistency within each factor as indicated by adequate but not strong Cronbach alpha. Although a small number of items loaded onto each factor could have contributed to this, it is worthwhile to note that some items excluded in the final scale were well-known determinants of HIV risk in this population, including age-disparate sexual relationships. Despite their theoretical relation to HIV risk, these items were dropped in the standard factor analysis mainly due to cross-loadings, which aimed to maintain the minimal number of items that can characterize the underlying vulnerability structure of sexually active AGYW. Further analyses of the scale using larger samples with greater variation in the responses would strengthen the study findings. Lastly, potential social desirability and reporting bias especially with regards to sexual history and violence may have led to misclassification of exposure status among AGYW. Despite these limitations, psychometric analysis results provided support for construct validity of the sale and sub-scales, which not only deepens the understanding of the latent structure of sexual health vulnerability among AGYW, but also provides a practical tool for stratifying HIV risk among AGYW in Tanzania. This sexual health and HIV risk stratification scale helps to advance efforts to reach this population as it is designed specifically for sexually active AGYW aged 15–24 years including the out-of-school adolescents below age 18. Using a minimum set of key HIV vulnerability items, the scale allows us to rapidly identify those who are at high risk of HIV acquisition and channel them to appropriate high-impact prevention-intervention programs, effectively reducing their advance in HIV risk as they mature from adolescence to adulthood.

## Conclusions

The sexual health and HIV risk stratification scale adds value to the growing body of HIV vulnerability measures targeting AGYW including the Adolescent Girls Vulnerability Index and the Vulnerable Girls scale [18,19,20]. Built on existing evidence, this risk stratification scale ascertained the latent structure of AGYW’s vulnerability and identified three main factors characterizing the underlying construct of vulnerability in AGYW. Moreover, it represents one of the few tools available to quantify AGYW’s sexual health vulnerability through observable items. This sexual health and HIV risk stratification scale is also unique in that it specifically targets those who are out of school due to either school drop-out or lack of access to education. As data have consistently shown, AGYW with limited education are at higher risk of HIV acquisition due to increased social and economic vulnerabilities [38]. There are over 5,000,000 adolescent girls and young women in Tanzania [58]. Fortunately, most are not at significant risk for HIV. In 2019, funding for HIV prevention programs is increasingly in jeopardy, necessitating increasingly specific, evidence-based, and cost-efficient responses. Moving forward necessitates short, focused tools as presented here to rapidly describe the heterogeneity in HIV risks among AGYW and characterize those at highest risk in order to inform program implementation.

## Supporting information

S1 Table Items in the original Sauti vAGYW HIV risk questionnaire. (DOCX)
