# Supplementary material for: Characterizing a sexual health and HIV risk stratification scale for sexually active adolescent girls and young women (AGYW) in Tanzania
Source: PLoS One. 2021 Mar 18;16(3):e0248153. doi: 10.1371/journal.pone.0248153 (PMC7971553; doi:10.1371/journal.pone.0248153)
Supplement: S1 Table — (DOCX) [file pone.0248153.s001.docx]

**Appendix**

**S1 Table. Items in the original Sauti vAGYW HIV risk questionnaire**

| **DOMAIN 1: SEXUAL BEHAVIOR** | | |
| --- | --- | --- |
| **Variables** | **Question** | **Response** |
| Sexual history | Have you ever had sex? If yes, have you had only vaginal sex, anal sex or both? We define sex as vaginal or anal intercourse | 1. Yes, vaginal and anal sex (3 points) 2. Yes, anal sex only (3 points) 3. Yes, vaginal sex only (2 points) 4. Never had vaginal or anal sex (0 points) |
| Age at coital debut | How old were you when you first had sex? | 1. ≤15 years old (3 points) 2. 16-18 years old (2 points) 3. >18 years old (0 points) |
| Recent sex | Have you had sex in the past 12 months? | 1. Yes (3 points) 2. No (0 points) |
| Condom use last three vaginal sex | Thinking about the last three times you had vaginal sex, how many of those times did you use a condom? | 1. None (3 points) 2. Once (3 points) 3. Twice (2 points) 4. All three times (0 points) 5. I don’t know (3 points) |
| Condom use last three anal sex | Thinking about the last three times you had anal sex, how many of those times did you use a condom? | 1. None (3 points) 2. Once (3 points) 3. Twice (2 points) 4. All three times (0 points) 5. I don’t know (3 points) 6. Never had anal sex (0 points) |
| Multiple sexual partners | In the last 12 months, have you had more than one sexual partner within the 30-day period | 1. Yes, over 3 sexual partners (3 points) 2. Yes, 2 sexual partners (2 points) 3. Yes, but don’t know how many (3 points) 4. No (0 points) 5. Don’t know (3 points) |
| Transactional sex | Have you ever had sex with anyone because he provided you or you expected that he would provide you with gifts, help you to pay for things, or help you in other ways? | 1. Yes (3 points) 2. No (0 points) 3. Refused (2 points) |
| Pregnancy history | Have you ever been pregnant? If yes, what age were you when you were first pregnant? | 1. Yes, <15 years old (3 points) 2. Yes, 15-17 years old (2 points) 3. Yes, 18-20years old (1 points) 4. Yes, >20 years old (0 points) 5. Yes, but don’t know age when first pregnant (1 point) 6. Never been pregnant (0 point) |
| Modern contraception use | Are you currently using any form of modern contraception? | 1. Yes (2 points) 2. No (3 points) |
| Condom use | If you are using modern contraception, do you also use a condom? | 1. Uses contraception but never a condom (2 points) 2. Uses contraception but only sometimes a condom (2 points) 3. Uses contraception and always uses a condom (0 points) |
| Age-disparate sexual relationships | What was the biggest age difference between you and a sexual partner? | 1. >10 years (3 points) 2. 6-10 years (2 points) 3. 3-5 years (1 point) 4. Less than 3 years (0 point) 5. Don’t know (3 points) |
| Partner’s HIV status | Do you know the HIV status of your current sexual partner(s)? | 1. At least one HIV+ partner (3 points) 2. All HIV- (0 points) 3. Don’t know (3 points) |
| **DOMAIN 2: SEXUAL AND GENDER VIOLENCE** | | |
| **Variables** | **Question** | **Response** |
| Sexual violence | At any time in your life, as a child or as an adult, have you ever experienced sexual violence? If yes, how often have you experienced this kind of sexual violence? Sexual violence is defined as any physical sexual act that is perpetrated against your will (this includes, for example, vaginal or anal penetration, digital penetration, and oral sex). | 1. Yes, >3 times (3 points) 2. Yes, 1-2 times (2 points) 3. Yes, once (1 point) 4. Never experienced sexual violence |
| Physical violence | Has anyone ever used their hands or an object to hurt you physically? This might include pushing, shaking, slapping, punching with a first, throwing with something, kicking, dragging, beating, chocking, burning, or threatening to attack with a knife gun or weapon. | 1. Yes (3 points) 2. No (0 points) 3. Don’t know (2 points) |
| **DOMAIN 3: IMPOVERISHMENT** | | |
| **Variables** | **Question** | **Response** |
| Food insecurity | In the past 30 days, have you gone to sleep at night hungry because there was no food? | 1. Yes, often (3 points) 2. Yes, sometimes (2 points) 3. Yes, rarely (1 point) 4. Yes, but don’t know how often (1 point) 5. Never experienced (0 point) |
| Child-headed household | How old is the head of your household? | 1. <15 or >70 years old (3 points) 2. 15-18 years old (2 points) 3. 19-24 years old (1 point) 4. 24-69 years old (0 point) |
| Marital status | Are you currently married or living together with a man as if married? | 1. No (0 point) 2. Yes, <18 years old (3 points) 3. Yes, 18 old and older (1 point) |
| **DOMAIN 4: ISOLATION** | | |
| **Variables** | **Question** | **Response** |
| Adult support | Is there an adult in your household or community to whom you can go for emotional and/or financial support? | 1. No (3 points) 2. Yes, but only emotional support (2 points) 3. Yes, but financial support (2 points) 4. Yes, both emotional and financial support (0 point) |
| Community participation | Are you a member of any social group which meets at least two times per month? | 1. No (3 points) 2. Yes (0 point) |
| **DOMAIN 5: SCHOOLING AND LITERACY** | | |
| **Variables** | **Question** | **Response** |
| Education | Have you ever attended school? If yes, what is the highest grade/form/year that you have completed | 1. Never attended school (3 points) 2. Yes, but did not complete primary school (3 points) 3. Yes, completed primary school (2 points) 4. Yes, but did not complete secondary school (1 point) 5. Yes, completed secondary school (0 point) 6. Yes, but don’t know highest grade completed (1 point) |
